# The Human Disease Ontology 2022 update

**DOI:** 10.1093/nar/gkab1063

**Published:** 2021-11-10

**Authors:** Lynn M Schriml, James B Munro, Mike Schor, Dustin Olley, Carrie McCracken, Victor Felix, J Allen Baron, Rebecca Jackson, Susan M Bello, Cynthia Bearer, Richard Lichenstein, Katharine Bisordi, Nicole Campion Dialo, Michelle Giglio, Carol Greene

**Affiliations:** University of Maryland School of Medicine, Institute for Genome Sciences, Baltimore, MD, USA; University of Maryland School of Medicine, Institute for Genome Sciences, Baltimore, MD, USA; University of Maryland School of Medicine, Institute for Genome Sciences, Baltimore, MD, USA; University of Maryland School of Medicine, Institute for Genome Sciences, Baltimore, MD, USA; University of Maryland School of Medicine, Institute for Genome Sciences, Baltimore, MD, USA; University of Maryland School of Medicine, Institute for Genome Sciences, Baltimore, MD, USA; University of Maryland School of Medicine, Institute for Genome Sciences, Baltimore, MD, USA; Bend Informatics LLC, Bend, OR, USA; Mouse Genome Informatics, The Jackson Laboratory, Bar Harbor, ME, USA; Case Western Reserve University, Cleveland, OH, USA; University of Maryland School of Medicine, Baltimore, MD, USA; University of Maryland School of Medicine, Baltimore, MD, USA; Internal Medicine Resident, PGY-1, Johns Hopkins Bayview Medical Center, USA; University of Maryland School of Medicine, Institute for Genome Sciences, Baltimore, MD, USA; University of Maryland School of Medicine, Baltimore, MD, USA

## Abstract

The Human Disease Ontology (DO) (www.disease-ontology.org) database, has significantly expanded the disease content and enhanced our userbase and website since the DO’s 2018 Nucleic Acids Research DATABASE issue paper. Conservatively, based on available resource statistics, terms from the DO have been annotated to over 1.5 million biomedical data elements and citations, a 10× increase in the past 5 years. The DO, funded as a NHGRI Genomic Resource, plays a key role in disease knowledge organization, representation, and standardization, serving as a reference framework for multiscale biomedical data integration and analysis across thousands of clinical, biomedical and computational research projects and genomic resources around the world. This update reports on the addition of 1,793 new disease terms, a 14% increase of textual definitions and the integration of 22 137 new SubClassOf axioms defining disease to disease connections representing the DO’s complex disease classification. The DO’s updated website provides multifaceted etiology searching, enhanced documentation and educational resources.

## Graphical Abstract

**Graphical Abstract:**
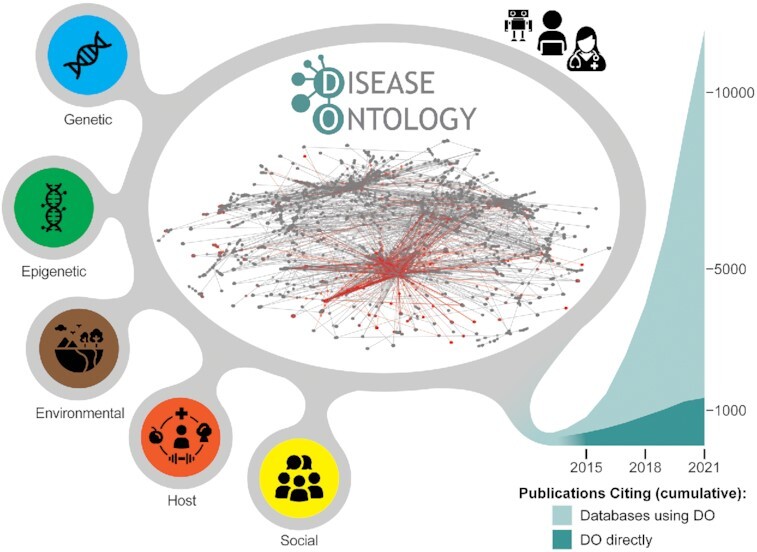
The Human Disease Ontology (DO) integrates disease features and etiological factors to describe disease complexity. The DO is extensively cited and integrated into highly cited databases.

## INTRODUCTION

Broadly useful genomic resources provide rapid access to collected knowledge in human-readable and computation-ready formats, thus breaking down interoperability barriers by harmonized data representations through the integration of semantic standards. Hence, building a standardized way of linking related genomic knowledge between resources makes disease-related data more Findable, Accessible, Interoperable, and Reusable (FAIR) (1). For example, the Alliance of Genome Resources (AGR) (2) has developed a unified model organism research platform that facilitates linkages between genes, alleles, human disease models, and human genes represented in the Model Organism Databases (3–9), utilizing ontologies and controlled vocabularies (Human Disease Ontology (DO), www.disease-ontology.org), Online Mendelian Inheritance in Man (OMIM), Mammalian Phenotype Ontology (MP), Human Phenotype Ontology (HPO) and UBERON (10–14)). Among these ontology resources, the DO plays a central role in classifying >10 800 rare, common and complex human diseases and is utilized by thousands of clinical, biomedical and computational researchers and genomic resources around the world. The DO’s terms, disease classification and mechanistic models have made the DO the de facto standard for disease etiology. The DO innovates interoperability between the DO and other ontologies through the integration of logical definitions formulated via ontology imports to define disease features and etiology.

The DO seeks to describe the breadth and complexity of human disease and to provide a stable framework for advanced analysis. Expansion of the DO’s content and disease models further advances our understanding of disease mechanisms, strengthens the disease information ecosystem and promotes the dissemination of knowledge that is responsive to new discoveries and paradigm shifts and that integrates basis science and clinical medicine. The breadth, number and diversity of the DO’s use cases continues to expand substantially each year, as the DO is utilized to explore genomic disease associations, and to facilitate development of effective health informatics tools that guide diagnostics and to develop disease-phenotype and disease-drug association predictions. Since our previous Nucleic Acids Research (NAR) DATABASE issue publication (10), we have made significant improvements and advances to our database.

### The DO’s CONTENT and WEBSITE EXPANSION

Here, we report on the significant enhancements to the DO database since 2018, including the expansion of the DO’s disease terms, logical definition content and the integration of additional ontology imports. We also report enhancements to the DO website that enable multifaceted data exploration and expanded outreach materials and activities.

### DO content (terms, axioms and imports) updates

We have augmented the DO’s content, including disease terms, Relation Ontology (RO) relations (15), and ontology imports, to support new data models. The 100th GitHub Release of the Human Disease Ontology (v2021-08-17) included 10 862 disease terms, with 76% (8312) of the disease terms defined by textual definitions. This release represents the addition of 1793 new disease terms and a 14% increase of textual definitions. Disease terms have been added fairly evenly across the DO classification hierarchy. Disease terms previously classified as asserted children of ‘genetic disease’ have been reclassified to be both asserted ‘syndrome’ child terms and inferred ‘genetic disease’ child terms. Notable new terms include SARS-CoV-2 related diseases and over 1000 OMIM genetic disease subtypes. Given the importance of OMIM as the gold standard reference for human genetic diseases and the relation of these disease terms to human genes, integrating and maintaining OMIM cross-references in the DO is a high priority set of tasks. These tasks include: refining and maintaining the relations between OMIM phenotypic series and DO terms; identifying cases where changes in OMIM result in a change in the disease attached to a specific OMIM ID; removing obsolete OMIM IDs from the DO; and adding new OMIM IDs to the DO as OMIM adds additional phenotypes and refines their OMIM phenotypic series representations.

The DO’s expert curators have researched and written over 8880 textual definitions (to date) following specific design patterns to capture the most pertinent information and document their associated 12 739 definition sources, identifying new knowledge from authoritative sources (e.g. OMIM, Genetics Home Reference, Gene Reviews, Genetic and Rare Diseases Information Center (GARD)) (16–18). The type of ‘source’ has been documented through an expansion of the Evidence and Conclusion Ontology (ECO), through the development of 11 new ECO codes under the parent term: ‘curator inference from authoritative source’ to specify if the definition source was a book, database, dictionary (e.g. MedlinePlus, Merriam-Webster, Oxford), encyclopedia (Britannica, MedlinePlus, Wikipedia) or a journal publication (19). ECO codes are programmatically assigned to definition sources as part of the DO release process, with each DO release given a unique, persistent digital identifier, enabling future analysis based on specific DO releases. The DO’s 100th Release includes 35 984 clinical vocabulary cross references, with new Medical Subject Headings (MeSH), NCI thesaurus and SNOMED CT terms (20–23) identified through the DO’s bi-annual Unified Medical Language System (UMLS) (24) update and with Orphanet (25), OMIM and GARD cross references manually added during curation of new DO terms. The DO’s integration of widely accepted clinical vocabulary standards enhances the DO’s value for linking data compiled within resources. The DO is a domain ontology and a semantic disease classification, is intended to be reasoned computationally, is formally structured and serves as a disease nomenclature and disease term harmonization resource supporting development of over 50 application ontologies.

Yearly, a number of complex genetic diseases are reviewed by the DO’s Clinical team to discern the complexity of genetic predispositions and revise their DO classifications. Terms reviewed since our previous NAR publication include amyloidosis, Ebstein's anomaly, hypertrophic cardiomyopathy, conformational diseases (prions), tuberous sclerosis, fetal alcohol syndrome, inherited metabolic disorders, DiGeorge/velocardiofacial syndrome/Deletion 22 syndrome, Chromosome 22q11.2 deletion syndrome, Alport syndrome, diabetes and asthma. Review of lysosomal storage diseases is ongoing. Additionally, the content of the DO has been enhanced to support usage of British English aliases in the Clinical Interpretation of Variants in Cancer (CIViC) database, through the incorporation of British synonyms into the ontology. We identified ∼30 commonly used clinical terms that have different British synonyms (e.g. diarrhea vs diarrhoea, hemorrhage versus haemorrhage), adding novel term pairs to the HPO British English dictionary. We utilized SPARQL Protocol and RDF Query Language (SPARQL) queries and a ROBOT tool (26) pipeline to programmatically add British synonyms to ∼150 DO terms (https://github.com/obophenotype/human-phenotype-ontology/blob/master/src/ontology/ hpo_british_english_dictionary.csv). The pipeline has been added to the DO’s Makefile to run each time we build a new DO release, thus creating British English synonyms for new DO terms. For example, the DO term ‘neonatal anemia’ would have the synonym ‘neonatal anaemia’ added as part of the DO’s build process. The current DO file includes additional RO relation terms. The RO term, ‘disease has feature’ is utilized to define when a disease, such as cataracts, is a feature of another disease. Previously, axioms defining anatomical locations of a disease were defined using the RO term ‘located in’ which has been replaced with the RO term ‘disease has location’ to align with RO revisions.

### Axioms

The DO’s clinical utility has been enhanced through the integration of the diverse and interconnected etiological genetic risk factors and environmental drivers of disease, to better represent complex disease by linking DO terms with those of related biomedical ontologies via highly structured design patterns. Here, we report on the growing number of inferred-classifications, empowered by the addition of a significant number of new SubClassOf axioms. The DO has established a novel complex disease classification, by defining disease to disease connections through the integration of 22 137 new SubClassOf Axioms, representing a significant increase from the 3612 SubClassOf Axioms reported in 2018. The DO contains these logical definitions (axioms) to describe relevant disease drivers, constructed with specific RO terms, as a Web Ontology Language (OWL) object property, to create a restriction between a DO term and another Open Biological and Biomedical Ontology (OBO) Foundry ontology term. For example, carcinoma is defined with an Equivalent Class axiom (‘cell type cancer’ and ‘derives from’ some ‘epithelial cell’) defining the cell type with Cell Ontology terms (27). Thus the relatedness of each organ system cancer with an epithelial cell of origin (e.g. trachea carcinoma and colorectal carcinoma) is defined via an ‘inferred’ parentage to carcinoma. Defining these relationships in the DO enables enhanced querying to identify DO terms sharing a common disease driver. Through this method, we are creating a taxonomy of linked diseases and allowing the user to investigate indirect links between diseases. The addition of highly structured design patterns for a comprehensive set of axioms linking DO and other biomedical ontologies will enable powerful queries exploring diseases with common etiological mechanisms, thus facilitating a more in-depth understanding of complex disease.

### Cross references

As our understanding of human disease changes and evolves over time it is necessary to review and revise disease entity relationships between different disease resources. In the DO, terms from many different disease resources are integrated as database cross-references (xrefs) to DO terms. We introduced the use of Simple Knowledge Organization System (SKOS) mapping terms (https://www.w3.org/TR/skos-reference/) (28) (skos:narrowMatch, skos:exactMatch and skos:broadMatch) to express mapping relationships and reconcile differences between the DO’s cross-references. SKOS mappings enable the DO to distinguish and define differences in ‘lumping and splitting’ of terms between vocabularies, with the skos:broadMatch utilized where the DO concept is broad and the xref is narrow (represents subtypes), and the skos:narrowMatch utilized where the DO concept is narrow (DO represents subtypes) and the xref is broad.

### DO’s ontology trees

The DO’s OBO and OWL trees provide a multi-parentage view of disease classification. The DO utilizes the ELK reasoner supporting the OWL 2 EL profile (https://www.w3.org/TR/owl2-profiles/). The DO’s OWL tree enables exploration of disease via the DO’s imports, thus enabling exploration of disease through related biomedical information (cancer, anatomical location, cell type of origin); environmental driver (infectious agents, chemicals, exposures, food allergies); genetic drivers (susceptibility traits, sequence variants); and clinical variants (phenotypes and symptoms). These novel views of disease provide a unique perspective, such as exploring related diseases or disease mechanisms through the lens of common disease drivers (genetic risk factors, biological, chemical or ecological factors), phenotypes, symptoms, mode of inheritance, or infectious agent.

### Classifications

Integration of up-to-date disease nomenclature refined by disease re-classifications has further enhanced the DO’s disease representation. The DO’s disease etiology-based classification has evolved to be responsive to what is more useful for our userbase, to provide an anatomical view of disease and to model environmental drivers of complex genetic diseases. These include the classification of pediatric cancers of acute lymphoblastic leukemia, B-lymphoblastic leukemia/lymphoma and gliomas to include the WHO’s molecular subtypes. This collaborative effort involved working with ClinGen and CIViC and integrating ICD-O (https://seer.cancer.gov/icd-o-3/) cross-reference mappings for pediatric cancers (29,30). A complete review and revision of the sarcoma branch was guided by Dr. Radoslav Davidovic at the Center for Multidisciplinary Research, Institute of Nuclear Sciences VINA, Belgrade, Serbia. The DO team, working with the Immune Epitope Database (IEDB) (31), has reviewed and integrated hypersensitivity, immune and inflammation related diseases. The DO team has also updated the nomenclature of benign neoplasms to incorporate a revised naming system and revamped the classification for Alzheimer's disease, early-onset and late-onset Parkinson's disease (coordinated with Mouse Genome Informatics (MGI) and OMIM). The DO’s genetic disease classification has been augmented with the addition of mechanistic data models (multi-genic, mode of inheritance, symptom and complex disease). This work has made it clear that reliable and accurate incorporation of entities from disease resources into the DO requires both a thorough understanding of disease concepts, coordination and co-development of guidelines and insight into the nuances of representation of these concepts by various resources. Only through careful review and consultation with these resources can we provide an integrated view of disease in the DO.

### Modeling complex disease etiology

**Figure 1.**
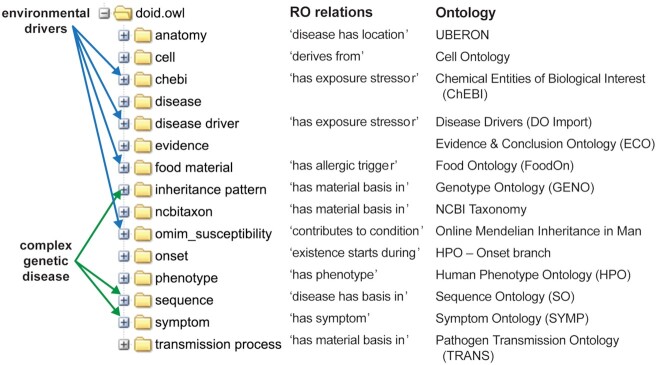
DO imports for defining environmental drivers and complex genetic disease etiology. The DO’s doid.owl tree integrates terms from 15 other biomedical ontologies to define features and etiology of human diseases (11,13,14,19,27,34–37, GENO: http://obofoundry.org/ontology/geno, NCBITaxon ontology: http://www.obofoundry.org/ontology/ncbitaxon.html; the Symptom, Pathogen Transmission, & Disease Drivers Ontologies are part of the DO project). Axioms are defined by pairing an ontology relation term and one or more ontology terms, such as ‘transmitted by’ some (‘droplet spread transmission’ or ‘airborne transmission’) for COVID-19.

Representation of the complexity of genetic etiology and environmental drivers of disease within the ontological structure of the DO presents a framework for developing a differential diagnosis resource. Beyond monogenic diseases, clinical diagnosis is challenged by the complexity of etiologies for multifactorial genetic diseases. The complexity of etiologies for differential diagnosis challenges established bioinformatic knowledge captured in biomedical ontologies. Classification of complex disease necessitates an evolution towards precise disease etiology captured through clinically-defined semantic assertions. To address the challenges of representing this clinical complexity, the DO project has developed a complex genetic disease model to drive the restructuring of the DO’s knowledge (Figure 1). As knowledge grows on how interactions between genetic and environmental factors lead to human disease, there is a need to incorporate genetic and environmental information into the DO. The pleiotropy of genetic diseases and the multi-organ impact of environmental factors further challenges the ontological representation of complex clinical knowledge.

### Imports and automated integration

The DO has enhanced identification of related diseases and enabled querying of diseases by related biomedical data with the inclusion of a suite of ontology imports. The DO integrates non-disease ontology terms to define connections between diseases, defined by logical axioms. For example, querying the axiom (‘has phenotype’ some ‘Abnormal immunoglobulin level’) retrieves 27 autoimmune-related disease terms. These logical assertions define relationships by connecting diseases through their shared attributes and allows viewing of inferred relationships between diseases to build a more complex multi-parental disease to disease classification. Utilizing ontology imports enables exploration of disease terms through their related cell of origin, anatomical location, symptoms, phenotypes, genetic or environmental risk factors. The DO’s import files are generated with each DO release using the ROBOT ‘extract’ command, which syncs the import file with their source ontologies (https://disease-ontology.org/resources/DO_Imports). The subset of ontology terms utilized from each import is defined by curated term lists. To maintain a reasonable size, only part of each ontology import file that is relevant to DO disease axioms are imported. The DO’s custom import term lists (see our GitHub import directory: https://github.com/DiseaseOntology/HumanDiseaseOntology/tree/main/src/ontology/imports) are updated and aligned to their source ontologies as part of the DO’s monthly data release. To date, the DO integrates 15 import files: anatomy, cell types, chemicals, (age of) onset, disease drivers, evidence codes, food material, inheritance pattern, omim_susceptibilty (genetic risk factors), phenotypes, sequence (structural and functional variants), symptoms, taxonomy and transmission methods (see Figure 1). The onset import is extracted from the HPO and the omim_susceptibility import is maintained by the DO team. More recent work has focused on the addition of disease drivers to define environmental factors that contribute to disease etiology, such as chemical exposures, nutritional deficiencies, mother to child smoking or alcohol exposure as mechanisms of disease. A subset of these are ‘environmental stressors’, a term in the Exposure Ontology (ExO) (32) and adopted into the Environmental conditions, treatments and exposures ontology (ECTO) (https://github.com/EnvironmentOntology/environmental-exposure-ontology). We have created and continue to develop a new application ontology (CC0 license), Disease Drivers Ontology (DISDRIV), to capture the environmental stressors, as adding the specific stressors themselves is out of scope for ExO and ECTO, which plan to represent the upper level terms only. The DISDRIV ontology is available in GitHub (https://github.com/DiseaseOntology/DiseaseDriversOntology).

### The DO’s website

**Figure 2.**
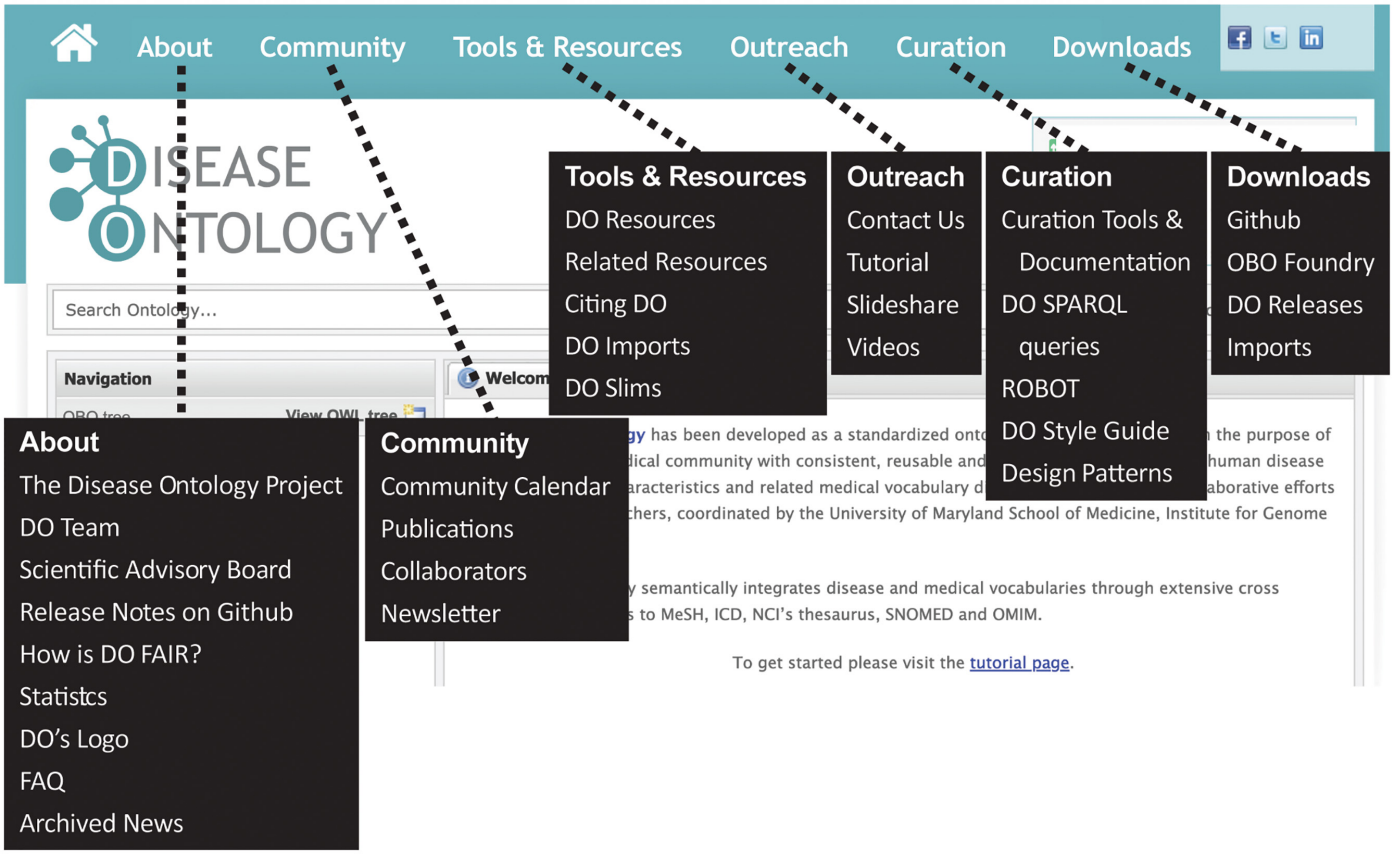
The DO website. Providing educational resources, multifaceted querying of OBO and OWL trees.

The DO’s website has been updated with new features, including new Community, Curation, Documentation, and Outreach pages (Figure 2). A new OWL tree view has been added to the DO website, based on the ‘doid.owl’ file, which enables searches that include any of the DO’s import files (e.g. cell types, anatomy, inheritance). Users can, for example, retrieve diseases that are located in the same body site (anatomical location) or retrieve all diseases that share a common mode of inheritance (e.g. autosomal recessive inheritance). The DO website provides publicly available documentation of curation methods, lists and links to the DO’s contributors and documented users, shares services offered via the DO’s website, documents peer-reviewed publications, and provides access to the DO’s educational videos on the DO’s YouTube channel (https://www.youtube.com/c/DiseaseontologyOrgDOID), a quarterly newsletter and the DO’s Tweets. Enhanced curation documentation includes: ‘DO SPARQL queries’, ‘ROBOT (automated QC, data release)’, ‘Design Patterns – Inheritance’, ‘DO Ticket Review Steps’, ‘DO merges FAQ’ and ‘How is DO FAIR’. The updated DO website includes monthly release notes and quarterly updated statistics for assessing usage and richness of content, including ‘Publications Citing the Disease Ontology Each Year’, ‘Disease Ontology Terms and Definitions by Release’, ‘Disease Ontology Branch Counts’ and ‘Disease Ontology Cross-References. The DO’s Community >> Publications page documents the DO’s ever expanding use cases and applications. The DO serves as the disease nomenclature resource across more than 280 biomedical resources and over 50 biomedical ontologies identified through the mining of the DO’s PubMed citations. The DO team has recently identified, via Scopus, more than 1300 DO project citations, a significant increase from the ∼700 reported in February 2021. The two ontology tree views provide multi-parentage views of the DO’s disease classification and enable exploration of disease through related biomedical information. The OBO tree presents diseases while the OWL tree presents diseases and imports. For example, to explore the etiology of ‘cancer’ users can examine anatomical location, cell type of origin; environmental drivers (infectious agents, chemicals, exposures, food allergies) and/or genetic risk factors (susceptibility traits, sequence variants) along with clinical variants (phenotypes and symptoms). This enables novel views of disease, for example, through the lens of common symptoms or the mode of inheritance, or infectious agent. Multifaceted searching enables exploration of the DO’s axioms through the Advanced Search option on the DO’s website (www.disease-ontology.org). Users can explore disease metadata by submitting queries for Name (disease name), Synonym, Definition, Subset (e.g. DO_cancer_slim or DO_AGR_slim), DOID, Alternate ID, Xrefs (from clinical vocabularies: OMIM, NCIthesaurus, ICD, SNOMED, GARD, Orphanet) or Relation. The recently added Relation option support querying of the DO’s axioms via RO relation terms: ‘adjacent to’, ‘derives from’, ‘disease has basis in’, ‘has allergic trigger’, ‘has material basis in’, ‘has origin’, ‘has symptom’, ‘has phenotype’, ‘disease has location’ or ‘transmitted by’. The Outreach section of the DO website includes three updated tutorials on the DO project, website navigation and exploring the DO’s OBO & OWL trees. DO slide decks developed for outreach presentations are available via SlideShare and figshare (https://figshare.com/account/home#/projects/124345). We have developed DO YouTube educational videos to demonstrate basic and advanced search functions of the DO website and to highlight DO use cases. These include: ‘What is an ontology?’; ‘Searching the Human Disease Ontology website’; ‘How is the Human Disease Ontology FAIR?’; ‘Clinical applications of the Human Disease Ontology’; ‘Mining disease information via imports: Connecting disease-related information’; ‘How the Human Disease Ontology is used for drug studies’; and ‘Cancer resources and tools utilizing the Human Disease Ontology’. The DO’s YouTube videos are available on the DO’s YouTube channel (https://www.youtube.com/c/DiseaseontologyOrgDOID) and as a playlist (‘The Human Disease Ontology)’ on the International Society for Biocuration (ISB) (https://www.youtube.com/channel/UCNLZMHYSuWSIjoOinpAxo_Q/playlists) and Biomedical Ontology World (https://www.youtube.com/channel/UCUT0MwXxAFnekhsSJVmHTJw/playlists) YouTube channels. The DO project actively tracks user requested updates or new term requests via the DO’s GitHub open-access repository (https://github.com/DiseaseOntology/HumanDiseaseOntology/tree/master/src/ontology). Since 2015, the DO team has fielded and resolved over 895 user requests to integrate new genetic disease subtypes, novel cancer variants and to include newly defined rare diseases. The DO project produces, with each release, subsets (‘slims’) of the DO ontology for specific use cases. These subset files are available in the DO’s GitHub. Subsets are created for specific use cases (e.g. AGR, FlyBase, MGI) or to facilitate querying the DO (e.g. NCIthesaurus cancer terms, rare disease). The newest bespoke DO slims include the ‘DO_RAD_slim’ created for the RadoNorm project (https://www.radonorm.eu/), an EU project focused on reducing the risks of radon and naturally occurring radioactive material (NORM) exposure, and ‘DO_GXD_slim’ created for the Gene eXpression Database (33).

## FUTURE DIRECTIONS

Modeling and defining the environmental components of complex diseases will expand the DO’s repertoire for differential diagnosis exploration. Expanding our understanding of the underpinnings and impact of genetic variation will enhance the DO’s multifaceted view of human disease etiology. We plan to enhance the DO’s dissemination through social media and expanded outreach opportunities.
